# MHC I of the Great Reed Warbler Promotes a Flat Peptide Binding Mode

**DOI:** 10.1111/imm.70015

**Published:** 2025-07-11

**Authors:** Raminta Venskutonytė, Sven Kjellström, Emily Amelia O'Connor, Helena Westerdahl, Karin Lindkvist‐Petersson

**Affiliations:** ^1^ Experimental Medical Science, Medical Structural Biology BMC C13, Lund University Lund Sweden; ^2^ LINXS ‐ Institute of Advanced Neutron and X‐Ray Science Lund Sweden; ^3^ Swedish National Infrastructure for Biological Mass Spectrometry‐ BioMS Lund Sweden; ^4^ Molecular Ecology and Evolution Lab, Department of Biology Lund Sweden

**Keywords:** antigens/peptides/epitopes, genomics, MHC, structural biology/crystallography

## Abstract

The major histocompatibility complex (MHC) plays a key role in pathogen recognition as part of the adaptive immune system. MHC I gene copy numbers in birds of the order Passeriformes (songbirds) are substantially larger compared to other birds. MHC I diversity and antigen presentation have been carefully characterised in chicken *Gallus gallus* of the order Galliformes; chickens express few MHC I genes and often present antigens that bulge out of the peptide binding cleft. This observation raises the question of whether MHC I presents antigens in a similar way in species with many MHC genes? Here, we present the X‐ray structure of MHC I from the great reed warbler *Acrocephalus arundinaceus* (Acar3) a long‐distance migratory songbird. Structural analysis shows that MHC I binds the antigen in a flat conformation due to a sequentially well‐conserved restriction point, acting like a pair of tweezers, within the peptide binding grove, created by Arg97 and Arg155. This more stringent antigen presentation by Acar MHC I molecules may partly explain the high MHC gene copy numbers seen in the great reed warbler.

## Graphical abstract

In this article we present the X‐ray structure of MHC I from the great reed warbler *Acrocephalus arundinaceus* (Acar3) a long‐distance migratory songbird. Structural analysis in combination with genetic analyses shows that MHC I binds the peptide antigen in a flat conformation due to a sequentially well‐conserved restriction point, acting like a pair of tweezers, within the peptide binding grove, created by arginine's. This more stringent antigen presentation by Acar MHC I molecules may partly explain the high MHC gene copy numbers seen in the great reed warbler.

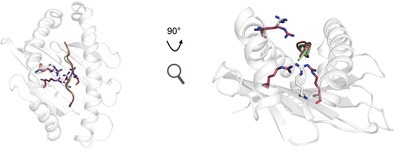

## 1 Introduction

Antigen presentation by major histocompatibility complex class I (MHC I) is a central mechanism of adaptive immunity. MHC I molecules are expressed on the surface of most nucleated cell types and present peptides (self or foreign antigens) to T cell receptors (TCR) on the surface of CD8^+^ T cells. MHC I molecules consist of two polypeptide chains, the α‐chain (including the α_1_, α_2_ and α_3_ domains, encoded by exons 2–4) and the β_2_‐microglobulin domain (β2m). The α_1_ and α_2_ domains create a peptide binding groove (PBG), containing six distinct binding pockets A–F, that can accommodate peptides consisting of typically 8–10 amino acids [1]. The N‐ and C‐terminus of the peptide are anchored within the opposite ends of the PBG, while the middle part of the peptides commonly bulges out of the PBG [2].

The chicken *Gallus gallus* (order Galliformes) has been an important model organism in the early studies of the adaptive immune system in vertebrates and has remained the key model species in avian immunology [3, 4]. Chicken has a compact MHC genomic region with two MHC I and two MHC IIB genes [5]. In contrast to chicken, songbirds, which belong to the largest and most recently derived bird order Passeriformes, have an extended MHC genomic region that is similar in size to the 4.0 million base pair MHC region in humans. The extended MHC region in songbirds has arrays of tandemly organised MHC I and MHC IIB genes, and the long‐distance migratory songbird, the great reed warbler *Acrocephalus arundinaceus* , has 15 MHC I genes and 56 MHC IIB genes [6]. There is a substantial MHC I gene copy number variation in great reed warblers, both between individuals and between haplotypes within individuals [7, 8]. The MHC I genes in the great reed warbler are highly polymorphic, and the highest genetic diversity is seen in the PBG where positively selected sites are maintained by balancing selection [7, 9]. A large proportion of the MHC I alleles in the great reed warbler genome are expressed, and the number of expressed MHC I alleles increases linearly with the number of MHC I gene copies in the genome [10]. These differences between chicken and songbirds raise the question: why do great reed warblers express many more MHC I genes than chicken?

According to theory, an optimal antigen presenting repertoire of the MHC molecules in an individual should present antigens from all pathogens the individual will be exposed to during its lifetime, while avoiding immune reactions to self‐peptides [11, 12, 13]. Therefore, individuals in high pathogen areas can be expected to have a broader antigen presenting repertoire of their MHC molecules compared with individuals in low pathogen areas. In line with this reasoning, previous research has shown that songbird species in high pathogen areas have higher MHC diversity than species in low pathogen areas [14, 15]. However, the antigen presenting repertoire of the MHC molecules within an individual is not only dependent on MHC diversity (the number of MHC genes and the heterozygosity per MHC gene) and overlap in antigen binding repertoire between different MHC molecules, but also on the total antigen binding repertoire of each MHC molecule [16, 17, 18]. In chicken, a wide range of different antigen binding repertoires is seen: fastidious MHC I molecules (e.g., BF2*1901 [19] and BF2*0401 [20]) present few peptides, whereas promiscuous MHC I molecules (e.g., BF2*0201 and BF2*1401 [18]) present many peptides [3]. Songbirds, irrespective of pathogen selection pressure, often maintain much higher MHC I diversity compared with other birds [21, 22, 23, 24, 25], and one explanation for the high MHC diversity could be MHC I molecules with a more stringent binding.

Here we report a structural analysis of MHC I from a long‐distance migratory songbird, the great reed warbler, in complex with peptide from the intracellular bacteria *Legionella*, verified to exist in gut microbiomes from passerines. The structure reveals important molecular determinants of the peptide binding groove, which result in a flat binding mode of the peptide. Based on these and previous results, we argue that this type of peptide binding, as opposed to the commonly seen more flexible peptide binding from other species, requires a larger number of MHC I alleles, hence higher MHC diversity, to ensure a satisfactory broad antigen presentation repertoire within an individual.

## 2 Results

### 2.1 X‐Ray Structure of MHC I Contains an Eight Amino Acids Long Peptide

**FIGURE 1:**
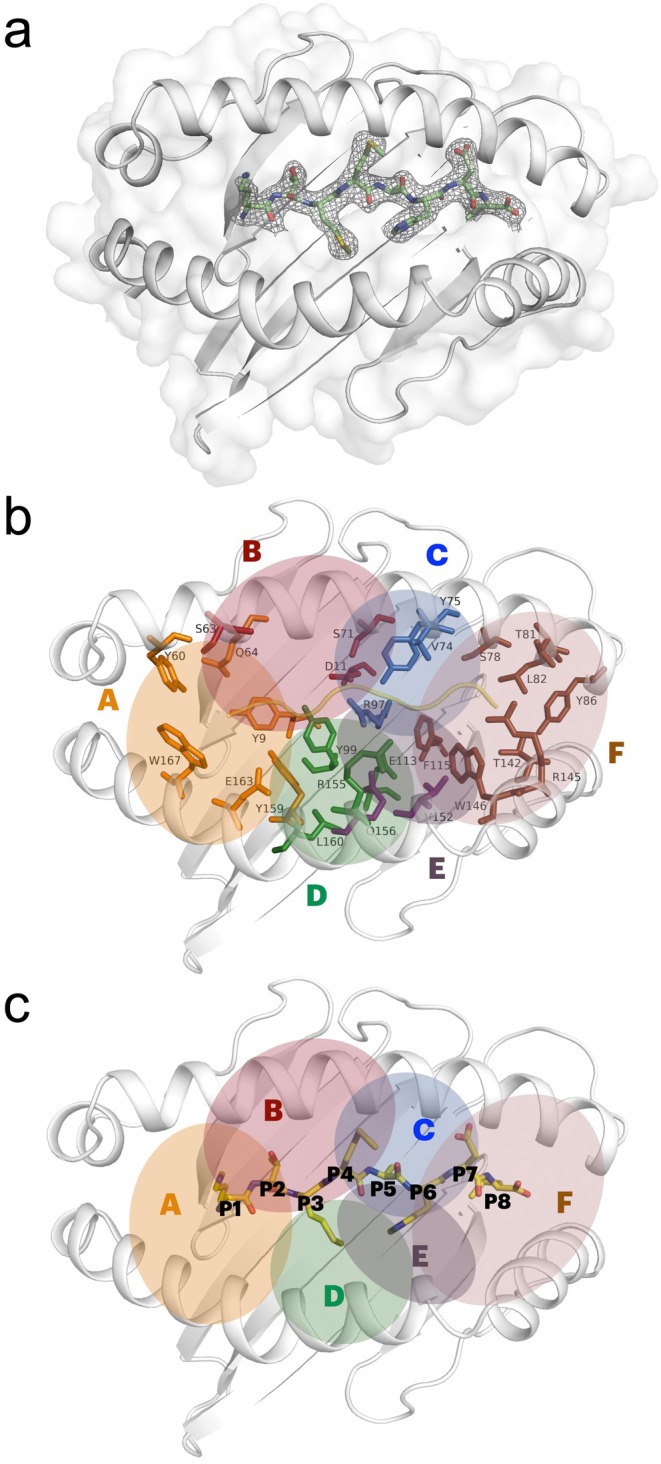
X‐ray structure of Acar3 shows an 8‐mer peptide bound to PBG. (a) KTM peptide in the Acar3 PBG with 2fo‐fc density at 1 sigma, carved around the peptide. (b) Acar3 PBG with different pockets indicated and amino acids in each pocket shown as sticks, and the peptide as cartoon. (c) Acar3 PBG with different pockets indicated and with the peptide (yellow) displayed in sticks.

We have previously studied the three‐dimensional structure of MHC I from the great reed warbler (Acar 3) in complex with two different 9‐mer peptides, YMTLQAVTF (abbreviated YMT) and MTMITPPTF (abbreviated MTM) that were selected based on a stability assay [26]. In the YMT structure, a methionine at position 2 (P2) is the N‐terminal anchor, while in the MTM structure a methionine at position 3 (P3) anchors the peptide, resulting in a register shift for the latter peptide suggesting that methionine is a conserved anchor residue for Acar3. To further clarify the importance of methionine as an N‐terminal anchor, the structure of Acar3 was refolded in the presence of a third peptide KTMMQAHDL and determined by X‐ray crystallography at 2.15 Å resolution (Table S1). Overall, the X‐ray structure of Acar3 in complex with the peptide shows the canonical peptide binding mode, with the peptide placed between the α‐helixes in the peptide binding groove (PBG) (Figure S1). The structure displayed an unambiguous electron density map for the peptide, and when modelling the peptide chain in the electron density map, it became clear that a 9‐mer peptide cannot be accommodated and that the peptide bound in fact is an 8‐mer, lacking a large side chain in the middle (Figure 1a). To confirm the identity of this 8‐mer, the peptide sample was analysed with mass spectrometry (Figure S2), which revealed that the sample indeed contains small amounts of 8‐mer impurities (< 5%). These included a peptide without the C‐terminal leucine (KTMMQAHD) and a peptide without a glutamine at P5 (KTMMAHDL). It was clear from the electron density that the latter peptide was bound to the Acar3 (Figure 1a). Since Acar3 was refolded in vitro with a commercially synthesised peptide, it is apparent that Acar3 has a strong preference for binding to the 8‐mer KTMMAHDL (abbreviated here after KTM‐peptide) instead of the 9‐mer KTMMQAHDL, which comprised the majority of the sample. The PBG is characterised by the typical six distinct binding pockets A–F (Figure 1b) [1]. Unlike the structures with YTM and MTM peptides, both having methionine as an N‐terminal anchor, pocket B is now occupied by a threonine (in position 2, Figure 1c), while the C‐terminal leucine is in pocket F (Figure 1c).

### 2.2 The Peptide Forms an Extensive Interaction Network Within the PBG

**FIGURE 2:**
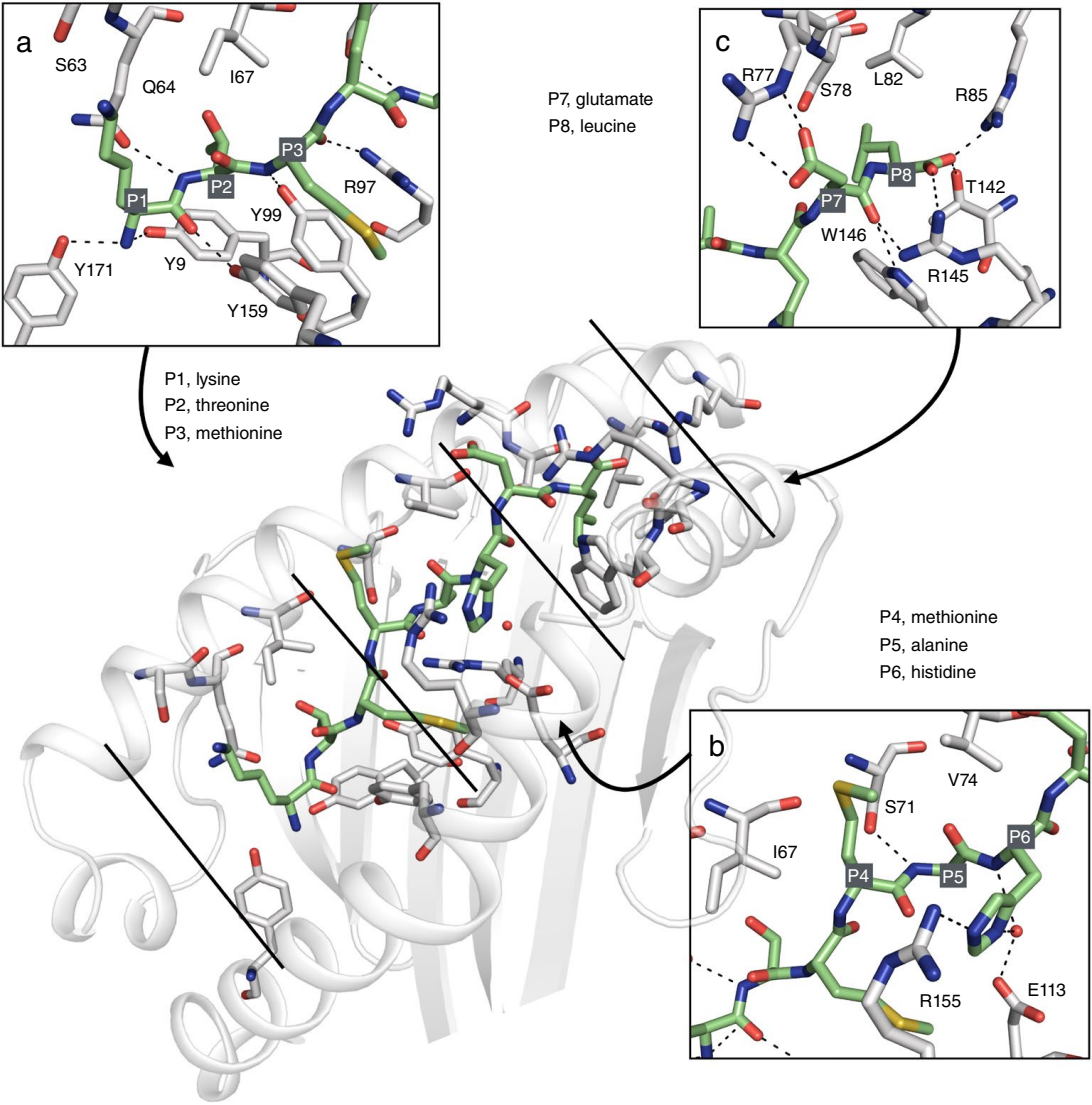
Acar3 has many atomic contacts with the KTM peptide. (a) Close‐up of the N‐terminal part of the peptide. (b) Close‐up of the middle part of the peptide. (c) Close‐up of the C‐terminal part of the peptide. The peptide (green) is shown in sticks and H‐bond interactions shown as dashed lines.

The first residue of the peptide, a lysine, is well accommodated in pocket A and the N‐terminal nitrogen forms hydrogen bonds to the sidechains of Tyr9 and Tyr171, while the backbone carbonyl forms an H‐bond to Tyr159 (Figure 2a). It is not possible to evaluate the exact conformation of the side chain of the lysine, but it points towards the sidechain of Ser63 and could potentially form a hydrogen bond depending on the rotamer. All these interactions by the lysine form a strong anchoring of P1 (Figure 2a). The next amino acid is threonine at P2 occupying pocket B and anchoring the N‐terminus of the peptide. Binding to MHC I is mediated by hydrogen bonding of the peptide's backbone nitrogen to the side chain of Gln64, and possibly it could also interact with Gln64 with its side chain oxygen (Figure 2a). Next, the two methionines, in the positions 3 and 4, are anchored in the PBG by backbone interactions to the side chains of Tyr99 and Arg97 with Met3 (Figure 2a), while Met4 has no backbone mediated H‐bonds, but its side chain is accommodated between the two hydrophobic residues Ile67 and Val74 (Figure 2b). At P5, there is an alanine that is stabilised by a backbone interaction to the side chain of Ser71 (Figure 2b). The next residue in the peptide is histidine at P6, which forms a hydrogen bond to the side chain of Arg155 via its side chain nitrogen, and it also forms water mediated interactions to the side chain of Glu113 (Figure 2b). The final two amino acids in positions 7 and 8 form an extensive interaction network within pocket F. The aspartate at P7 forms a salt bridge to Arg77, as well as a hydrogen bond via its backbone carbonyl to the side chains of Trp146 and Arg145 (Figure 2c). The C‐terminal carboxyl of the last residue, a leucine, establishes contacts to the side chains of Arg85 and Thr142 (Figure 2c). Overall, the peptide can form 14 interactions to the PBG residues via its backbone, with only two residues (P4 and P6) lacking backbone mediated hydrogen bonding. In addition, there are three side chain and two water mediated contacts. Such an extensive interaction network suggests a high‐affinity binding to the PBG.

### 2.3 Peptides at Acar3 Display a Flat Binding Mode

**FIGURE 3:**
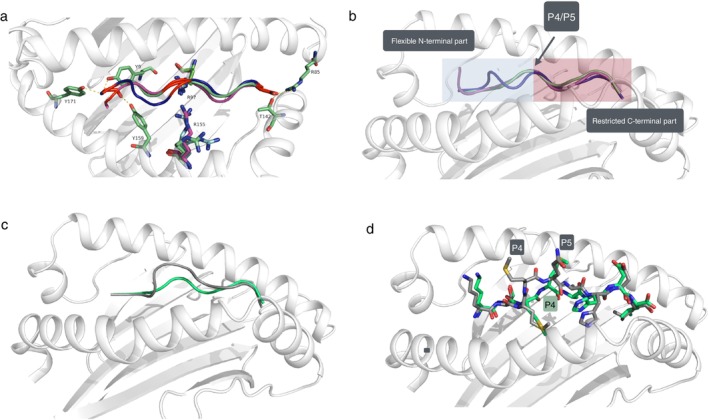
Acar3 peptides show a flat binding mode. (a) Overlay of the two 9‐mers, YMT (blue) and MTM (magenta) (PDB ID: 7QZI and 7QZJ, respectively) and an 8‐mer KTM peptide (green) shown as cartoon. The three anchor points at N‐terminus, the mid part and the C‐terminus of the peptides are coloured red. The amino acids at N‐ and C‐termini are shown as sticks for the Acar3‐KTM complex and Arg97 and Arg155 are shown as sticks for all three peptide complexes. (b) Overlay of the two 9‐mers YMT (blue) and MTM (magenta), and the KTM 8‐mer (green) with marked flexible N‐terminal and static C‐terminal part as well as the middle P4/P5 residue. (c) An overlay of the 9‐mer KTM complex generated by AlphaFold Server (grey) versus 8‐mer KTM experimental structure (green) shown in cartoon. (d) An overlay of the 9‐mer KTM complex generated by AlphaFold Server (grey) versus 8‐mer KTM experimental structure (green) shown in sticks.

In agreement with our earlier observations of the Acar3 binding mode of 9‐mer peptides (YMT and MTM) [26], Arg97 binding to the middle of the KTM peptide is a third determining anchor for the positioning of the peptides. In all three peptides studied for Acar3, hydrogen bonds are observed between the side chain of Arg97 and the backbone carbonyl of P3/P4 in the peptides (Figure 3a). Another conservation between the three structures is that both N‐and C‐terminal residues establish similar interactions: in pocket A, Tyr9, Tyr159 and Tyr171 with the N‐terminal residues, while the C‐terminal residues interact in an equivalent manner with Thr142 and Arg85 (Figure 3a). Based on the three available structures it can be concluded that despite sequence variations, the anchor sites of the termini of the peptides are conserved as well as the preference for adopting a flat binding mode (Figure 3a) [26]. Furthermore, an arginine (Arg155) located at helix 2 is folding over the peptides in the three structures (Figure 3a). In the KTM structure, Arg155 adopts a slightly different conformation compared to the previously published 9‐mer structures (YMT and MTM) to accommodate a larger residue at P6 (histidine vs. alanine/proline), and in addition Arg155 provides an H‐bond to the histidine side chain (Figure 2b). In the 9‐mer structures, Arg155 can bind to the backbone of the peptides as the residues at P7 are less bulky than a histidine and allow for Arg155 to reach even closer to the peptide. However, while Arg155 clearly can adapt to the sequence of the peptide, the presence of Arg97 at the bottom of the PBG and Arg155 facing the PBG from the top might in fact be a restriction point of the PBG to keep the peptide in the flat conformation (Figure 3a). This notion is further supported by the previously studied MTM 9‐mer peptide adopting a register shift, that is instead of having its P2 residue in pocket B, the P3 methionine is accommodated by pocket B, resulting in that the P1 residue is protruding from the PBG, and consequently the MTM peptide has an 8‐mer binding mode in the PBG, instead of creating the classical bulge (Figure 3b). In the KTM peptide, there is a threonine in P2 binding pocket B (as in the MTM‐peptide), assuring that threonine can fit into pocket B if that would have been favourable. Altogether, this suggests a preference for a flat peptide binding mode for Acar3. An explanation for this binding mode is likely the additional restriction site in the middle of the PBG, a putative peptide selectivity point created by Arg97 and Arg155 (Figure 3a). Interestingly, Arg97 and Arg155 are very well conserved among the MHC I in the great reed warbler, with 40% of 387 different MHC I exon 3 alleles having both arginines and only 5% of the Acar alleles having neither of the arginines (Table 1), suggesting that the flat binding mode is frequent among the MHC I molecules in the great reed warbler.

**TABLE 1:** Occurrences and frequencies of MHC‐I exon 3 alleles, with and without the arginines Arg97(95) and Arg155(152) in great reed warbler *Acrocephalus arundinaceus* (Acar 3) (Arg97 and Arg155; *N* = 387 alleles) and chicken *Gallus gallus* (Gaga) (Arg95 and Arg152, *N* = 52 alleles).

Arg97/	Arg155/	Nr Seqs	Frequency	Nr Seqs	Frequency
Arg95	Arg152	Acar	Acar	Gaga	Gaga
Y	Y	154	40%	1	2%
Y	N	133	34%	5	10%
N	Y	79	20%	20	38%
N	N	21	5%	26	50%

### 2.4 Acar3 Specific Features of the Pockets Allow Both 8‐Mer and 9‐Mer Peptides to Bind

In contrast to the classical peptide binding mode with the peptides bulging out of the PBG, Acar3 seems to have an additional restriction site (Arg97‐Arg155) favouring a flat binding mode (Figure 3a). Therefore, flexibility must be possible in other regions of Acar3 to accommodate 9‐mer or longer peptides. When overlaying the two 9‐mer structures with the 8‐mer complex, the backbone of the peptides aligns very well in the middle and in the C‐terminal parts of the peptide (the last five residues), while the N‐terminal parts of the peptide are more varied (Figure 3b). We have previously concluded that the adaptability of pocket A and the spaciousness of pocket B in Acar3 permit flexibility of the N‐terminal part of the peptides [26]. Hence, these features of pocket A and pocket B are possibly required to accommodate longer peptides, such as 9‐mers. This is supported by the fact that the threonine at P3 in the YMT‐peptide is creating a small bulge in the N‐terminal end when compared to MTM and KTM‐peptides (Figure 3b). Moreover, the importance of plasticity in the A/B pockets to accommodate longer peptides is also supported by the structural data of the MTM‐peptide, where instead of bulging out, the first residue is protruding from the PBG, creating a register shift, which essentially makes this 9‐mer bind as an 8‐mer, with the third residue acting as a N‐terminal anchor. Thus, flexibility in the binding mode to pocket A and pocket B is crucial to accommodate the preferred flat binding mode of Acar3 peptides (Figure 3a,b).

To investigate why Acar3 did not bind the 9‐mer KTM‐peptide, an AlphaFold structural model (AlphaFold Server (BETA) [27]) of the KTM 9‐mer peptide (KTMMQAHDL) bound to Acar3 was calculated (with interface predicted template modelling (ipTM) score of 0.92) (Figure 3c,d). In contrast to the experimental structure, the AlphaFold model resulted in the 9‐mer adopting a different and more classical‐like MHC I binding mode, with residues at P4 and P5 bulging out of the groove (Figure 3c,d). However, as Acar3 excluded the 9‐mer KTM peptide during the experimental refolding (although it was in great majority), a bulged binding mode must be unfavourable and have much lower affinity than the flat binding mode, supporting that Acar3 prefers peptides that bind in a flat conformation instead of creating the classical bulge.

### 2.5 Arginines Impose Flat Peptide Binding Mode in Chicken MHC I

**FIGURE 4:**
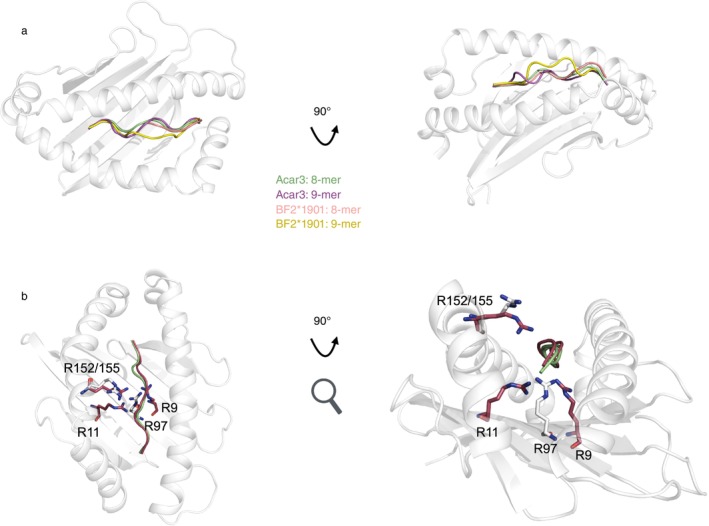
Structural comparison with chicken MHC peptide binding. (a) Cartoon representation of an overlay of the Acar3 KTM complex (green) and Acar3 YMT complex (PDB ID 7ZQI, magenta) with the chicken BF2*1901 8‐mer and 9‐mer complex (PDB IDs: 7WBG, salmon; 7WBI, yellow). (b) Overlay of the Acar3 KTM complex (green) with the chicken BF2*0401 8‐mer (PDB ID 6LHG, burgundy). Arginine anchors—Arg97/Arg155 in Acar3 and Arg9/Arg111/Arg152 in BF2*0401 are shown as white and burgundy sticks, respectively.

In contrast to the large number of MHC I genes expressed by the great reed warbler, chicken only expresses one major MHC I gene (BF2). Structural and sequential analysis of 24 chicken MHC I (BF2) structures presenting 8–11‐mers was executed (based on seven different alpha chains (MHC I alleles), Table 2). Eleven MHC I structures in complex with 8‐mer peptides (PDB ID: 6LHH, 6LHF, 6LHG [28], 7WBG [19], 6IRL, 6KX9, [29], 5YMW [30], 4G43, 4G42, 4E0R [20], 4D0D [18]), five with 9‐mers (4CVX, 4CW1 [18], 5YMV [30], 7WBI [19], 8Y74 [31]), five with 10‐mers (3BEW [32], 2YEZ, 4CVZ, 4D0B, 4D0C [18]) and three structures with 11‐mer peptides were analysed (5ACZ, 5ADO [33], 3BEV [32]). As expected, all the 8‐mers bound in the chicken MHC I displayed a rather flat conformation, as also seen for the great reed warbler, whereas almost all analysed 9‐mer peptides (and 10‐ and 11‐mers) bulge out from the PBG in the chicken MHC I, in contrast to the flat conformation seen for the great reed warbler. Three different chicken MHC I structures were available with both 8‐ and 9‐mers (BF2*1901, *0201 and *1201). Both BF2*1901 (PDB ID: 7WBG, 7WBI [19]) and BF2*0201 (PDB ID: 4D0D, 4CVX [17], 8Y74 [25]) displayed a flat binding of the 8‐mers whereas the 9‐mers adopted the classical bulged‐out conformation, which is in clear contrast to Acar3 (Figure 4a, Figure S3) [26]. Nonetheless, BF2*1201 is an interesting exception since both the 8‐mer and the 9‐mer peptides adopt flat conformations, where the 8‐mer has the classical N and C‐terminal anchor points whereas the 9‐mer has the C‐terminal residue protruding outside of the PBG. This is possible since Arg83, which interacts with the C‐terminal of the peptide, adopts a new conformation by flipping its side chain away from the PBG making space for the extra residue to be accommodated [30]. Moreover, BF2*1201 has an arginine (Arg152, in the corresponding position to the Arg155 in Acar3), while all other available chicken MHC I X‐ray structures in complex with 9‐mers or longer peptides have a glycine in this position, suggesting that this arginine might be a contributing factor for the flat peptide‐binding mode, in analogy to Arg155 in Acar3 (Table 2). Although the structurally studied chicken MHC I commonly lack an arginine in position 152, it is rather frequent in chicken MHC I alleles (40%) (Table 1, Table S2). In contrast, arginines in positions 95 (corresponding to Arg97 in Acar 3) and 152 are very rare (2%), possibly explaining why chicken 9‐mers still generally bulge out from the PBG, as they lack the third restriction point (Table 1). However, the BF2*0401 molecule, which was shown to only bind 8‐mers and is classified as a fastidious MHC I [18], has a similar third restriction point as Acar3. BF2*0401 has Arg152 but instead of an arginine at position 95, it has two arginines, at positions 9 and 111, binding to the peptide from the bottom of the PBG, displaying similar functions as Arg97 in Acar3 (Table 2). Here, Arg9 and Arg152 hydrogen bond to the backbone carbonyls of the peptide, while Arg111 hydrogen bonds to the side chain of the peptide, creating a similar middle restriction point as in Acar3 (Figure 4b) [20]. However, arginine in positions 9 and/or 111 is rather rare (11%) in chicken MHC I alleles (*N* = 85 alleles, with only six alleles having both, one allele only Arg111 and two alleles have only Arg9, exons 2–4, Table S2). Interestingly, Arg111 does not exist among the 387 great reed warbler MHC I alleles (Table 1). Taken together, MHC I's with a preference for 8‐mers (BF2*1501, BF2*0401) have a flat binding mode irrespective of arginines (Table 2), but MHC I with these arginines can create a restriction point which enforces a flat peptide binding mode in chicken MHC I for longer peptides (BF2*1201, Table 2), potentially contributing to a more stringent peptide selectivity.

**TABLE 2:** Great reed warbler *Acrocephalus arundinaceus (Acar3)* and chicken Gallus gallus (BF*) MHC I structures presenting 8‐ to 11‐mer peptides. The arginines (Arg97(95) and Arg155(152)) and (Arg9, Arg11 and Arg155(152)) marked as 1 or 0 depending on presence in the sequence.

Alpha chain	Construct PDB ID	Peptide length	Arg9	Arg97 (95)	Arg111	Arg155 (152)	Expression level
*Acar_3*	7QZI (YTM)	9	0	1	0	1	high
	7QZJ (MTM)	9	0	1	0	1	
	9QG8 (KTM)	8	0	1	0	1	
BF2*1501	6LHH	8	0	0	0	0	high
	6LHF	8	0	0	0	0	
	6KX9	8	0	0	0	0	
	6IRL	8	0	0	0	0	
BF2*1901	7WBI	9	0	0	1	0	high
	7WBG	8	0	0	1	0	
BF2*0401	4G43	8	1	0	1	1	high
	4G42	8	1	0	1	1	
	4E0R	8	1	0	1	1	
	6LHG	8	1	0	1	1	
BF2*1201	5YMW	8	0	0	0	1	high
	5YMV	9	0	0	0	1	
BF2*2101	3BEW	10	1	0	0	0	low
	2YEZ	10	1	0	0	0	
	4CVZ	10	1	0	0	0	
	4D0C	10	1	0	0	0	
	4D0B	10	1	0	0	0	
	5ACZ	11	1	0	0	0	
	5AD0	11	1	0	0	0	
	3BEV	11	1	0	0	0	
BF2*0201	4D0D	8	1	0	0	0	low
	8Y74	9	1	0	0	0	
	4CVX	9	1	0	0	0	
BF2*1401	4CW1	9	0	0	0	0	low

### 2.6 Closely Related Songbird Species Have Similar Proportions of Arg155 in MHC I

**FIGURE 5:**
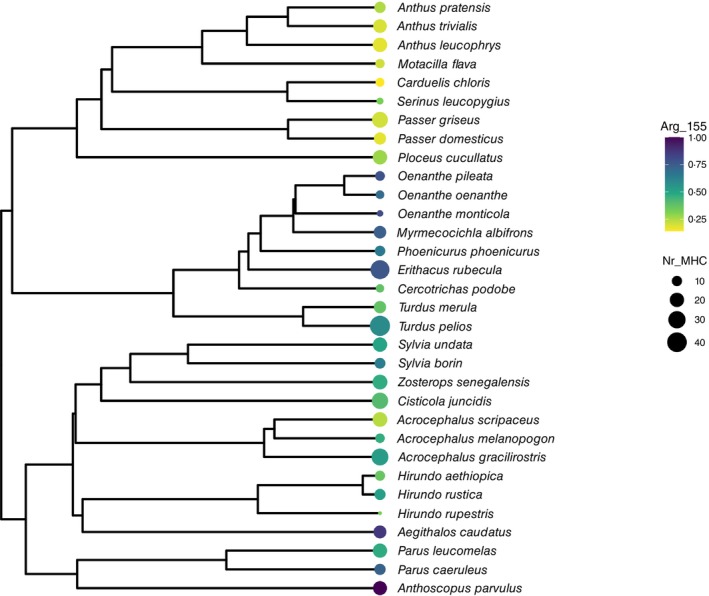
Phylogenetic reconstruction of 32 songbird species. The average MHC I diversity (size of the circle) and the proportion of Arg155 (colour scheme, low (yellow), moderate (green) high (blue)) per individual for each species is high‐lighted. Size of the tip symbols represents the mean MHC I diversity per species.

To investigate if the third restriction point, Arg97 and Arg155, is also common in MHC I alleles in other songbird species, and if the occurrence of these arginines explains the high MHC I diversity seen, we performed a phylogenetic comparative analysis using data from 32 representative species across Passerides that had a common ancestor 30 million years ago [14]. This data set was chosen because the study involved a standardised sampling and DNA sequencing approach that ensures the between‐species comparisons of MHC I diversity are robust. However, the section of exon 3 sequenced in this study did not include Arg97 and therefore only the presence of Arg155 in relation to MHC I diversity could be examined. The dataset consisted of 1311 MHC I exon 3 alleles from 81 individuals across 32 species. The proportion of MHC I alleles with Arg155 in songbirds had a high degree of phylogenetic signal with 83% of the variation in proportion of MHC I alleles with Arg155 across species being explained by phylogeny, showing that species within the same taxonomic groups tend to have a similar proportional representation of alleles with Arg155 (Figure 5, Table S3). The proportion of MHC I alleles with an arginine present at position 155 did not appear to influence MHC diversity in this dataset (posterior mode = 0.251, credible interval −0.454 to 1.086, MCMC derived P‐value = 0.21, Table S4). However, the lack of association from this initial test is likely due to the absence of Arg97 from this data. The mean percentage of Arg155 across the 32 songbird species was 48%, which is slightly lower than in the great reed warbler (60%) and slightly higher than in the chicken (40%) (Figure 5). Hence, future analyses including the whole exon 3 sequence are needed to properly evaluate the relationship between MHC I diversity and the presence of the third restriction point in other songbird species.

## 3 Discussion

The structural analyses of the MHC I from the great reed warbler, together with the sequential analyses, suggest that the Arg97 and Arg155 residues create an additional restriction point for Acar3 to ensure a flat binding mode that potentially contributes to a more stringent peptide selectivity. In fact, as most peptides bound to MHC I bulge out in the middle, they undergo dynamic changes upon TCR binding and are pushed deeper into the groove upon interaction with TCR [34]. This may also lower the affinity for the TCRs that can recognise them [35], as fewer structural features are available for recognition. This together suggests that the flat conformation seen for Acar3, without TCR bound, could represent a peptide that is already in a favourable conformation for TCR recognition but leading to lower overall affinity of the pMHC‐TCR complex. The stringent binding of peptides for Acar3 can therefore be one explanation for the high MHC I gene copy numbers in great reed warblers (Order Passeriformes) compared with non‐passerines. Hence, great reed warblers may need many different MHC I molecules per individual to satisfactorily present an adequate number of peptide antigens to activate a proper T cell response. Our phylogenetic analyses of 32 songbird species showed that the proportion of Arg155 in MHC I, one of the arginines in the third restriction point, has a strong phylogenetic signal, suggesting it is likely that songbirds within the same phylogenetic group as the great reed warbler, i.e., warblers, have similar proportions of Arg155. Although our preliminary analyses suggested a lack of positive association between the proportion of Arg155 and MHC I diversity across songbirds, the presence of both Arg97 and Arg155, the third restriction point, could be key to detecting this relationship since the presence of both arginines is frequent in the great reed warblers but rare in chicken. Interestingly an alternative third restriction point is observed in the chicken, as the role of Arg97 can be replaced by Arg9 and/or Arg111. In fact, chicken BF2*0401 has Arg9, Arg111 and Arg152 (corresponding to Arg155) and this seems to impose a flat binding mode, as BF2*0401 only binds 8‐mers [20]. A stringent peptide binding mode is not only related to a third central restriction point but is also influenced by the size of the PBG. Han and co‐workers compared two different chicken MHC I molecules that both can present 8‐mers, and proposed that the shallower and narrower PBG of the BF2*1901 results in fastidious peptide binding as fewer peptides can be accommodated with good affinity, while the BF*1501 displays a wider and a deeper PBG and therefore can bind a larger set of peptides [19]. The PBG of Acar3 is more like the BF2*1901 (shallow and narrow) and thus the suggested structural determinants for a more restricted binding may also apply for Acar3.

The 9‐mer peptide that was in majority in the experimental set up could most likely not be accommodated in the PBG of Acar3 since the fifth residue must bulge out of the PBG, which seems to be not permitted by Acar3. To accommodate 9‐mers in Acar3, either a register shift should occur (like in the MTM‐peptide where the first amino acid in the peptide sticks out of the PBG) or the N‐terminal part of the peptide should be able to fit into pocket A and B of the PBG (as seen for YMT 9‐mer peptide) [26]. The YMT peptide has glutamine in the fifth position, but still displays a flat binding mode, as the N‐terminal can be accommodated in pockets A/B. In contrast, for the KTM peptide, the pocket A/B can only accommodate three residues (instead of 4 as in YMT peptide) and therefore only the 8‐mer version of the KTM‐peptide could bind to the PBG. This suggests that the sequence of the peptide can also be a deciding factor if the 9‐mer can be accommodated since only the flat binding mode is possible for the middle and C‐terminal part, due to the third restriction point. Interestingly, the Acar3 8‐mer peptide sequence exists in a hypothetical protein from the intracellular bacteria *Legionella* (strain *Legionella* sp. *227*, bioRxiv [36]). The genus *Legionella* has been verified in bird gut microbiomes from passerines (KH Bodawatta, unpublished data) and considering the habitat preference of great reed warblers being close to water, exposure to bacteria of the genus *Legionella* would not be impossible, though yet to be confirmed.

The fastidious vs. promiscuous MHC I antigen presentation modes, which have been carefully investigated in chicken, correlate with their expression levels; the fastidious binding MHC I's show higher and the promiscuous MHC I's show lower expression levels in both chicken and human [18]. It was hypothesised that these binding modes by MHC I's may have evolved to present peptides from different types of pathogens. The fastidious and promiscuous MHC I's have also been described as specialists and generalists, respectively, where the latter would be more suited to fight common pathogens while the specialists would undergo a strong selection upon exposure to the novel pathogen [18]. This is an interesting perspective considering the great reed warbler MHC I's (Acars), as the overrepresentation of specialist MHC I alleles, as indicated by their stringent peptide binding mode, would suggest an advantage for great reed warblers in interactions with novel pathogens. Ability to handle novel pathogens is perhaps a selective advantage in the migratory lifestyle of great reed warblers since they encounter pathogens across a broad range of geographical regions; at their breeding site in the northern Palearctic, at several stop‐over sites during the Spring and Autumn migration, and finally at their wintering sites in sub‐Saharan Africa. Taken together, we suggest that the flat binding mode could provide an advantage for interactions with novel pathogens and this feature would then benefit pathogen detection and elimination, which is central to a successful migratory lifestyle.

## 4 Methods

### 4.1 Protein Production

Acar3 and β2m from Acar were prepared as previously described (Eltschkner et al., 2023). The functional MHC I was refolded by adding purified Acar3, β2m and a synthetic peptide KTMMQAHDL (Schafer‐N, Denmark) to the refolding buffer (100 mM Tris/HCl, pH 8.0, 400 mM L‐Arginine, 1 mM EDTA, 5 mM GSH, 0.5 mM GSSG). To determine Acar3 binding peptide sequences, a peptide library was screened for Acar3 binding using a scintillation proximity assay measuring the stability of the peptide‐Acar3 complexes [20]. First, 6.5 mg of Acar3, 6.5 mg of β2m and 5 mg of peptide were mixed into 500 mL of the refolding buffer in the evening. The next morning, 6.5 mg of Acar3 was added and then again in the evening of the same day the buffer was supplemented by another portion of 6.5 mg Acar3. After approximately 40 h of incubation in total, the buffer was dialysed against 5 L of 50 mM Tris pH 7.5, 150 mM NaCl, 0.5 mM EDTA. After around 3 h of dialysis the buffer was changed to 3 L of 50 mM Tris pH 7.5, 150 mM NaCl and dialysed overnight. After the dialysis, the sample was filtered, concentrated and subjected to size exclusion chromatography (Superdex 200 Increase 10/300 GL, Cytiva) in a buffer of 20 mM Tris pH 7.5, 150 mM NaCl. The peak fractions of the refolded complex were collected.

### 4.2 Mass‐Spectrometry

The purchased synthetic peptide was analysed with reversed phase chromatography coupled with a timsTOF HT instrument with standard DDA settings using a gradient of 44 min as previously described [37]. Database search was performed with PEAKS version X against the possible peptide sequences allowing oxidation of methionine and deamidation of glutamine. The total ion current consisted mostly of the KTMMQAHDL peptide and its modified forms. The degraded form of KTMMQAHDL, i.e., KTMMQAHD, and a version of the peptide without glutamine was also detected, i.e., KTMMAHDL. All extracted ion chromatograms were confirmed with MSMS analysis (data not shown).

### 4.3 Crystallisation and Structure Determination

Fractions containing the refolded protein after size exclusion chromatography were pooled and concentrated to 10 mg/mL. The protein was subjected to crystallisation using the vapour diffusion hanging drop method by mixing 1 μL of protein with 1 μL of reservoir solution. The crystallisation conditions were screened using a broad commercial crystallisation screen (Hampton research, USA). One condition yielded initial crystals, which were then further optimised by varying precipitant concentration and applying streak seeding. The crystal used for structure determination was obtained in a condition consisting of 28% PEG4000, 0.1 M MgCl_2_, and 0.1 M Tris/HCl pH 8.5 with seeding applied. The X‐ray diffraction data was collected at the BioMAX beamline [38]. The data was processed using XDS [39] and Aimless [40] within the CCP4 program suite [41] and the structure solution was obtained by molecular replacement using Phaser [42] within Phenix [43] using 7ZQJ as a search model. Then the model was rebuilt in Autobuild [44] within Phenix [43] and refined with phenix.refine [45]. The model was edited and inspected in Coot [46] in between the refinements. The structural figures were prepared using Pymol (Schrödinger). When examining the crystal packing, we were concerned if it could influence the peptide binding mode. Indeed, the PBG of the two symmetry‐related molecules is facing each other, and the aspartate in P7 of the peptide is 3.4 Å away from the Arg77 of the symmetry‐related molecule. However, the overall solvent channels between the symmetry‐related molecules are large enough, and the N‐terminal part of the peptide is mostly solvent, suggesting that the peptide binding mode is not affected by crystal packing.

### 4.4 Sequence Analyses in Great Reed Warbler and Chicken

For the great reed warbler, there is mainly information from MHC I exon 3 in GenBank, and we used all available exon 3 amino acid sequences (*N* = 387 unique MHC I exon 3 amino acid sequences in open reading frame) in downstream analyses [47]. Basic Local Alignment Search Tool (BLAST, https://blast.ncbi.nlm.nih.gov/Blast.cgi) was used when searching for available MHC I sequences in chicken, and MHC I sequences were retrieved using pBLAST with a representative MHC I exon 2–4 amino acid sequence. Chicken MHC I sequences were also retrieved using the IPD‐MHC database https://www.ebi.ac.uk/ipd/mhc/allele/list. Only exon 2–4 sequences in open reading frame were kept, after which alignment files with unique MHC I exons 2–4 and exon 3 amino acid sequences were prepared in AliView version 1.28 (chicken, *N* = 85 and *N* = 52 unique MHC I exons 2–4 and exon 3 amino acid sequences, respectively) [48].

### 4.5 Phylogenetic Analyses

Songbird MHC I exon 3 alleles, published in O'Connor et al. 2018 [14] (1311 alleles, 32 species, 1–3 individuals per species) were used to estimate the prevalence of Arg155 alleles. The section of the MHC I gene amplified by the primers used in O'Connor et al. 2018 did not include the position of Arg97 and therefore these analyses only examined the frequency of alleles with Arg155.

We quantified the phylogenetic signal in the proportion of MHC I alleles that contained Arg155 using Bayesian Phylogenetic Mixed Models (BPMM) implemented in the R package ‘MCMCglmm’ version 2.36 [49]. We fit intercept only models with the proportion of Arg155 alleles in each species as the response variable. A binomial error distribution was used. We included a phylogenetic relationship matrix as a random effect in our models. Using the subset tool on the Bird Tree website (http://birdtree.org/), we downloaded a sample 10 000 trees from the posterior distribution of the Ericson all‐species backbone tree for the 32 species required [48]. We created a maximum clade credibility consensus tree from these 10 000 trees using the R package ‘phangorn’ (version 2.12.1) [50]. This maximum clade credibility tree was used to create the phylogenetic relationship matrix in the model. The model was run 2 000 000 times with the first 1 000 000 iterations discarded as burn‐in and the results of every 1000 iterations saved. This resulted in a posterior sample of 1000 estimates. Parameter estimates were summarised using the posterior mode (PM) and 95% credible interval (CIs). Terms were considered statistically significant when both the 95% CIs did not span 0 and pMCMC values (the number of iterations greater or less than zero) were below 0.05 [49]. We specified inverse‐Wishart priors (V = 1, nu = 0.002) for all variance components in the model (residual variance and random effects). Model convergence was tested by repeating each analysis three times and examining the correspondence between chains in R using the ‘coda’ package version 0.16–1 (https://cran.r‐project.org/package=coda) by: (1) visually inspecting the traces of the MCMC posterior estimates and their overlap; (2) calculating the autocorrelation and effective sample size of the posterior distribution of each chain; and (3) using Gelman and Rubin's convergence diagnostic test, which compares within‐ and between‐chain variance using a potential scale reduction factor. Values higher than 1.1 indicate chains with poor convergence properties. All three approaches indicated that models converged. The phylogenetic signal was estimated by calculating the proportion of the total variance explained by phylogeny. This was estimated on the logit scale.

To investigate the relationship between the proportion of MHC I alleles with Arg155 and MHC diversity, we used a similar BPMM approach. The response variable was the mean number of MHC I alleles with a fixed effect of the proportion of MHC I alleles with Arg155. A Poisson error distribution was used. Species was included as a random effect to account for the non‐independence of estimates taken from multiple individuals from the same species. To account for the non‐independence of data due to species ancestry, we included a phylogenetic relationship matrix as a random effect in our models. All models converged. Full details of all model specifications and results can be found in Tables S3 and S4. Raw data and R scripts used for the BPMMs can be found in Dryad [47].

## Author Contributions

Designed the experiments: R.V. and K.L.‐P. (structural biology), H.W. (genetics). Conducted the experiments: R.V. (structural biology), S.K. (MS). Analysed the data: R.V. and K.L.‐P. (structural biology), S.K. (MS) and H.W., E.A.O. (genetics). R.V., H.W. and K.L.‐P. wrote the manuscript with contributions from all co‐authors.

## Conflicts of Interest

The authors declare no conflicts of interest.

## Supporting information


**Data S1.** Supporting Information.

## Data Availability

The data that support the findings of this study are openly available in a Dryad Digital Repository (DOI: 10.5061/dryad.cvdncjtg1) [47]. The protein structure has been submitted to the Protein Data Bank with access code PDB ID 9QG8.
