# Supplementary material for: MHC I of the Great Reed Warbler Promotes a Flat Peptide Binding Mode
Source: Immunology. 2025 Jul 11;176(4):508–19. doi: 10.1111/imm.70015 (PMC12583234; doi:10.1111/imm.70015)
Supplement: Supplementary file 1 — Data S1. Supporting Information. [file IMM-176-508-s001.docx]

**Supplemental data for**

**MHC I of the great reed warbler promotes a flat peptide binding mode**

Raminta Venskutonytė^1,2^, Sven Kjellström^3^, Emily Amelia O’Connor^4^, Helena Westerdahl^4,#^, Karin Lindkvist-Petersson^1,2,#,*^

^1^Experimental Medical Science, Medical Structural Biology, BMC C13, Lund University, SE-221 84 Lund, Sweden

^2^LINXS - Institute of Advanced Neutron and X-ray Science, Scheelevägen 19,

SE-223 70, Lund, Sweden

^3^ Swedish National Infrastructure for Biological Mass Spectrometry- BioMS, Lund, Sweden.

^4^Molecular Ecology and Evolution Lab, Department of Biology, Ecology Building, SE-223 62 Lund, Sweden.

^*^Corresponding author: Karin Lindkvist-Petersson, Experimental Medical Science, Medical Structural Biology, BMC C13, Lund University, SE-221 84 Lund, Sweden. Tel: +46734222786, Email: [Karin.Lindkvist@med.lu.se](mailto:Karin.Lindkvist@med.lu.se)

^#^shared last authors


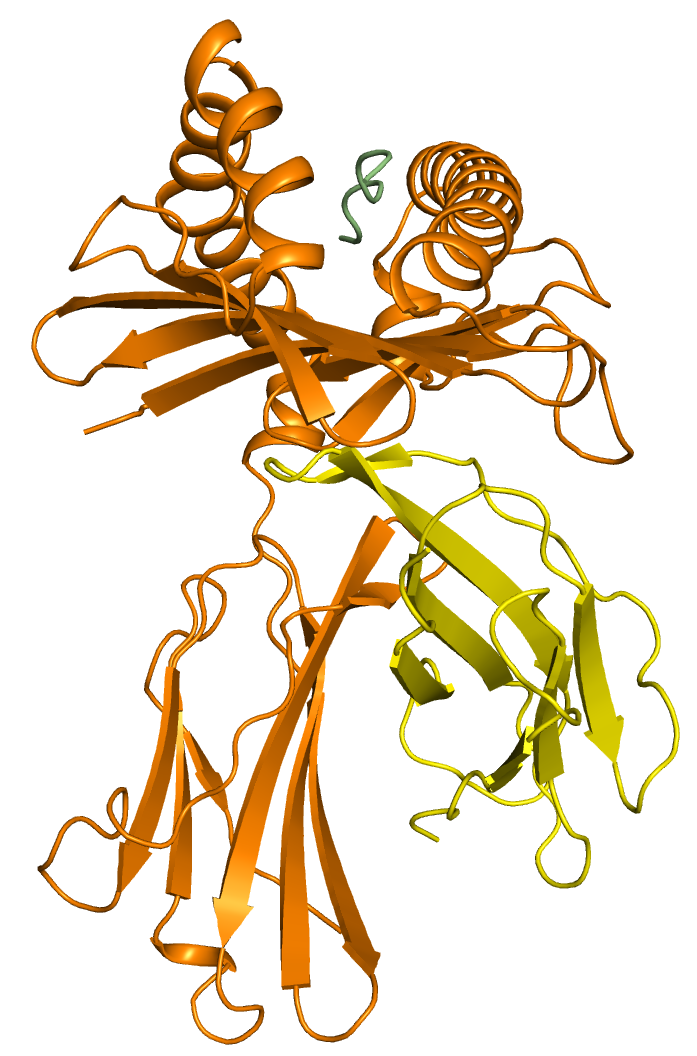


**Figure 1.** **Cartoon representation of the Acar3-KTM X-ray structure**. The alpha chain is shown in orange and the β2-microglobulin in yellow. The peptide in the PBG is shown in green.


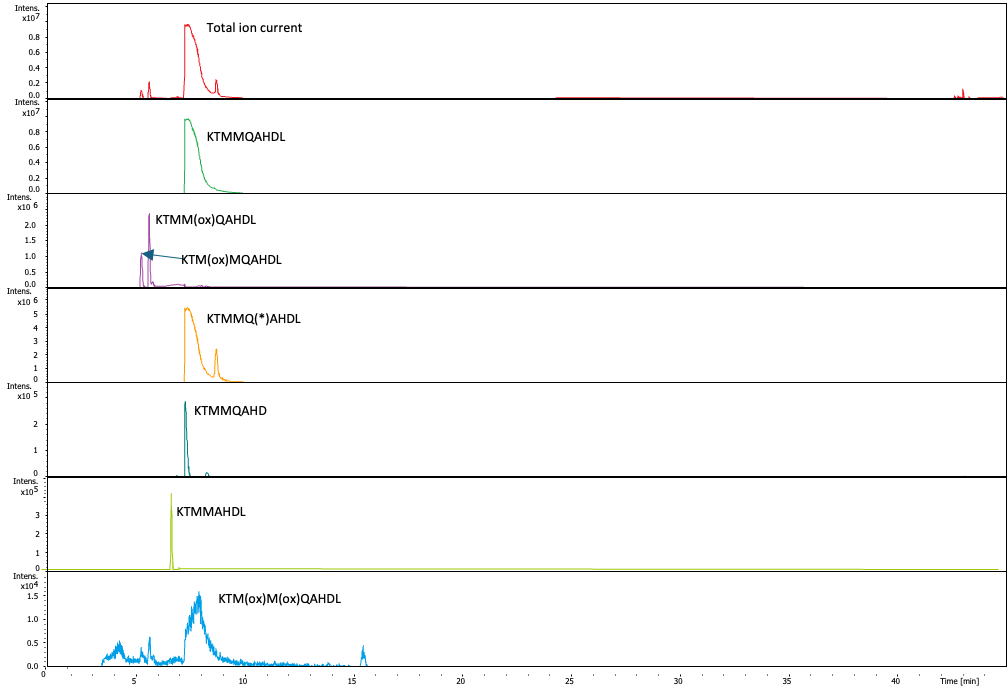


**Figure 2.** **Peptide analysis with reversed phase chromatography coupled with a tims TOF HT mass spectrometer**. The top panel shows total MS signal, and each panel below represents extracted separate peaks for different peptide species identified within the sample.


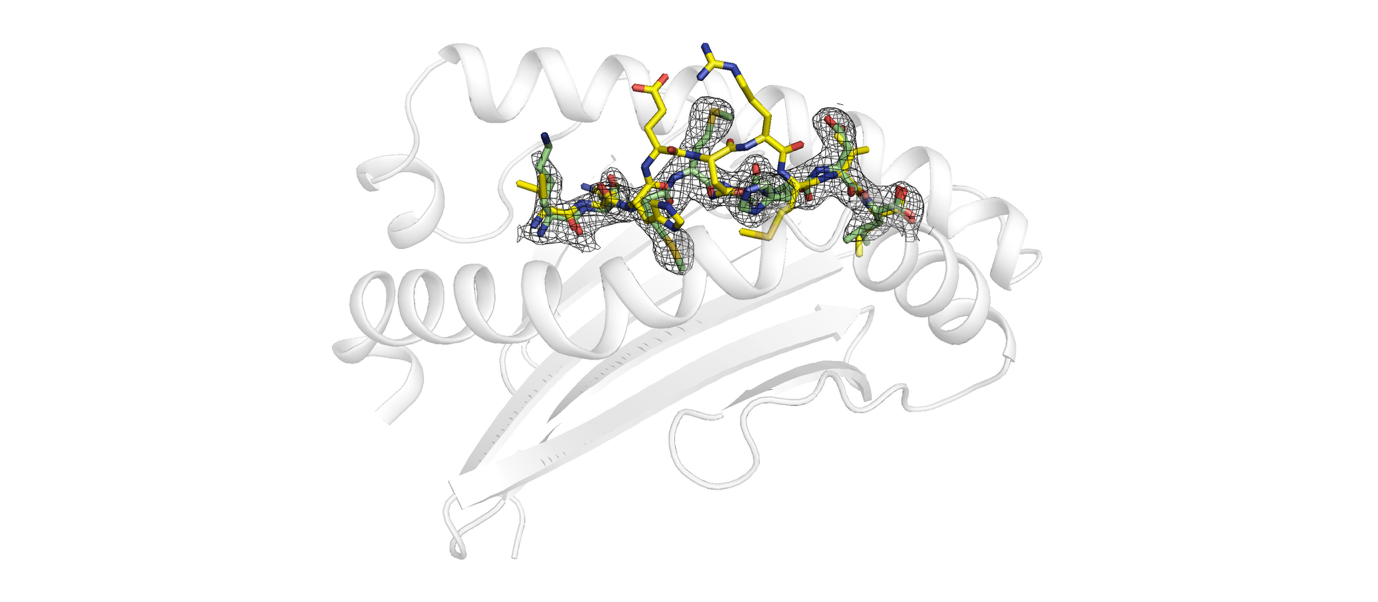
 **Figure 3.** Overlay of the Acar3 KTM complex (green) with electron density shown for the bound peptide with the chicken BF2*1901 9-mer complex (PDB ID: 7WBI, yellow).

**Table 1. Data collection and refinement statistics.**

| **Data collection** | **Acar3-KTM** |
| --- | --- |
| Space group | C 1 2 1 |
| Cell dimensions |  |
| *a, b, c* (Å) | 205.947, 49.038, 39.38 |
| α, β, γ (°) | 90, 94.69, 90 |
| Resolution (Å) | 47.7 - 2.15 (2.25 - 2.15) |
| *R_merge_* | 5.3 (62.1) |
| Mean *I/σI* | 10.7 (1.7) |
| CC 1/2 | 99.8 (75.3) |
| Completeness (%) | 99.6 (99.9) |
| Redundancy | 3.4 (3.4) |
| **Refinement** |  |
| No. reflections | 21416 |
| *R_work_/R_free_* | 20.56/26.57 |
| Number of non-hydrogen atoms | 3221 |
| ligands | 36 |
| solvent | 85 |
| protein residues | 380 |
| RMS (bonds) | 0.003 |
| RMS (angles) | 0.56 |
| Ramachandran favored (%) | 98.13 |
| Ramachandran allowed (%) | 1.87 |
| Ramachandran outliers (%) | 0.00 |
| Rotamer outliers (%) | 0.30 |
| Clashscore | 4.75 |
| Average B-factor | 53.16 |
| macromolecules | 53.07 |
| ligands | 66.72 |
| solvent | 50.50 |

**Highest resolution shell is shown in parentheses**

**Table 2**. Occurrences and frequencies of MHC-I alleles, with and without the arginines Arg97(95) and Arg155(152) in chicken *Gallus gallus* (Gaga) (Arg95 and Arg152, exon 2-4; N=85 alleles).

| **Arg97/**  **Arg95** | **Arg155/**  **Arg152** | **Nr Seqs**  **Gaga exon** | **Frequency**  **Gaga exon** |
| --- | --- | --- | --- |
| Y | Y | 6 | 7% |
| Y | N | 5 | 6% |
| N | Y | 37 | 44% |
| N | N | 37 | 44% |

**Table 3.** Phylogenetic signal in the proportion of Arg155 MHC-I alleles across species analysed using a BPMM implemented in the R package ‘MCMCglmm’. The error distribution used to model the response variable (Proportion of Arg155 alleles) was multinomial. Note that Phlyo H^2^ is calculated on the logit scale. Number of observations: 32; Number of species: 32.

| **Model** | **Fixed effects** | **Posterior mode (CI)** | **pMCMC** |
| --- | --- | --- | --- |
| Proportion of Arg155 alleles per species ~ 1, random=~phylogeny | Intercept | 0.937 (0.398, 1.427) | **<0.001** |
|  | **Random effects** | **Posterior mode (CI)** | **Phylo H^2^ (CI)** |
|  | Phylogenetic variance | 0.450 (0.169, 1.019) | 0.826 (0.429, 0.909) |
|  | Residual variance | 0.158 (0.057, 0.391) |  |

**Table 4.** Relationship between the number of MHC-I alleles and the proportion of MHC-I alleles with Arg155 in individuals across 32 species analysed using a BPMM implemented in the R package ‘MCMCglmm’. The error distribution used to model the response variable (Number of alleles per individual) was poisson. Number of observations: 81; Number of species: 32.

| **Model** | **Fixed effects** | **Posterior mode (CI)** | **pMCMC** |
| --- | --- | --- | --- |
| Number of alleles per individual ~ Proportion of Arg155 alleles per individual, random=~phylogeny+species | Intercept | 2.497 (1.950, 3.04) | **<0.001** |
|  | Proportion of Arg155 alleles | 0.251 (-0.454, 1.086) | 0.21 |
|  | **Random effects** | **Posterior mode (CI)** | **Phylo H^2^ (CI)** |
|  | Phylogenetic variance | 0.224 (0.073, 0.557) | 0.512 (0.199, 0.727) |
|  | Species variance | 0.237 (0.077, 0.467) |  |
|  | Residual variance | 0.062 (0.035, 0.097) |  |
